# Lnc NR2F1-AS1 Promotes Breast Cancer Metastasis by Targeting the MiR-25-3p/ZEB2 Axis

**DOI:** 10.7150/ijms.86969

**Published:** 2023-07-24

**Authors:** Duanyang Zhai, Yu Zhou, Xiaying Kuang, Fangyuan Shao, Tiantian Zhen, Ying Lin, Qing Wang, Nan Shao

**Affiliations:** 1 Breast Disease Center, The First Affiliated Hospital, Sun Yat-sen University, Guangzhou, China.; 2 Division of Vascular Surgery, National-Local Joint Engineering Laboratory of Vascular Disease Treatment, Engineering and Technology Center for Diagnosis and Treatment of Vascular Diseases, Guangdong Engineering Laboratoty of Diagnosis and Treatment of Vascular Disease, The First Affiliated Hospital, Sun Yat-sen University, Guangzhou, China.; 3 Cancer Center, Faculty of Health Sciences, University of Macau, Macau SAR, China.; 4 Department of Pathology, The First Affiliated Hospital, Sun Yat-sen University, Guangzhou, China.; 5 Department of Toxicology, School of Public Health, Sun Yat-sen University, Guangzhou, China.

**Keywords:** Lnc NR2F1-AS1, miR-25-3p, ZEB2, metastasis, breast cancer

## Abstract

**Background:** Long noncoding RNAs (lncRNAs) substantially affect tumor metastasis and are aberrantly expressed in various cancers. However, its role in breast cancer (BC) remains unclear.

**Methods:** A microarray assay of differentially expressed lncRNAs in epithelial-mesenchymal transition (EMT) and non-EMT cells was performed. The prognostic value of lnc NR2F1-AS1 expression in patients with BC was analyzed using The Cancer Genome Atlas database. Lnc NR2F1-AS1 expression levels in different BC cell lines were assessed using quantitative real-time PCR. The role of lnc NR2F1-AS1 in BC cell metastasis was investigated in vitro and in vivo. Dual luciferase reporter assay and RNA immunoprecipitation were performed to investigate the relationship between lnc NR2F1-AS1, miR-25-3p, and ZEB2.

**Results:** High levels of lnc NR2F1-AS1 were observed in BC cells undergoing EMT and were closely correlated with adverse prognosis in patients with BC. Lnc NR2F1-AS1 knockdown significantly inhibited BC cell migration, invasiveness in vitro, and metastasis in vivo. Mechanistically, lnc NR2F1-AS1 competitively binds to miR-25-3p to impede ZEB2 degradation, a positive EMT transcription factor in BC.

**Conclusions:** Our study revealed a novel lnc NR2F1-AS1/miR-25-3p/ZEB2 axis in BC metastasis and that lnc NR2F1-AS1 may serve as a potential therapeutic target for BC metastasis.

## Introduction

Breast cancer (BC) is the most prevalent cancer among women worldwide 1, 2. Although therapeutic attempts have primarily focused on preventing BC progression, the prognosis of patients with metastatic BC remains poor. Metastasis is a pivotal cause of high BC mortality and involves epithelial-to-mesenchymal transition (EMT), migration, invasion, and mesenchymal-to-epithelial transition (MET) 3. Therefore, the mechanisms triggering this process must be investigated.

Non-coding RNA was previously thought to be transcriptional noise. Accumulated evidence has revealed that non-coding RNA plays essential roles in various physiological and pathological processes 4-6. Long non-coding (lnc)RNA has lengths exceeding 200 nucleotides and can code proteins 7. Researchers worldwide have revealed that lncRNAs are modulated in tumors and are closely related to the progression of several solid cancers. The lncRNA MCM3AP-AS1 promotes hepatocellular carcinoma growth through the miR-194-5p/FOXA1 pathway. Moreover, the lncRNA MEG3 regulates melanoma growth, metastasis, and formation 8-10. In breast cancer, lnc NR2F1-AS1 acted as an oncogene and was associated with tumor recurrence in estrogen receptor (ER) positive breast cancers 11, 12. Previously, we found that lnc NR2F1-AS1 promoted BC angiogenesis by targeting the IGF-1/IGF-1R/ERK axis 13. However, the effects of lnc NR2F1-AS1 on BC metastasis regulation remain unclear.

In this study, we explored the biological functions, molecular mechanisms, and prognostic value of lnc NR2F1-AS1 in BC. Our results showed that lnc NR2F1-AS1 was significantly upregulated in BC cells, particularly in invasive BC cells, and correlated with poor outcomes in patients with BC. Inhibition of lnc NR2F1-AS1 resulted in decreased migration, invasion, and EMT capacities. In contrast, the overexpression of lnc NR2F1-AS1 showed contrasting results. Mechanistically, lnc NR2F1-AS1 acted as a competing endogenous RNA (ceRNA) for miR-25-3p to upregulate ZEB2 expression. Our data demonstrated that lnc NR2F1-AS1/miR-25-3p/ZEB2 axis is a promising therapeutic target for BC metastasis.

## Materials and Methods

### Clinical sample

Twenty-two pairs of triple negative breast cancer tissues and adjacent non-cancerous tissues were obtained from patients who received treatment at the First Affiliated Hospital of Sun Yat-sen University. This study was approved by the Medical Ethics Committee of the First Affiliated Hospital of Sun Yat-sen University. All enrolled patients signed a written informed consent form according to the relevant regulations. The tissues and matched adjacent-tumor controls were snap-frozen immediately in liquid nitrogen after being separated and stored at -80 °C before use.

### Bioinformatics

A KM plotter (http://kmplot.com/analysis/) was used to evaluate the survival prognosis of patients with BC from The Cancer Genome Atlas (TCGA). StarBase (http://starbase.sysu.edu.cn/) was used to predict the lncRNA-microRNA (miRNA) interactions, the miRNA target genes, and their correlations. Kyoto Encyclopedia of Genes and Genomes (KEGG) pathway genome enrichment analysis was conducted using R language.

### Animal model

A breast cancer metastasis assay was conducted in female nude mice (5-6 weeks old). All animal experiments were performed in accordance with the Guide for the Administration of Affairs Concerning Experimental Animals. Cells (1 × 10^6^) were injected into the tail vein of nude mice. After 3 weeks of inoculation, the lungs were removed, and metastatic nodules were counted. Whole lungs were removed, fixed in paraformaldehyde, embedded in paraffin, and stained with hematoxylin-eosin. The number of metastatic lung foci was calculated to evaluate the development of pulmonary metastases.

### Cell culture

The human breast epithelial cell line (MCF-10A) and breast cancer cell lines (MDA-MB-231, BT549, MCF-7, and ZR-75-1) were obtained from the American Type Culture Collection (ATCC; Rockville, MD, USA) and cultured in Dulbecco's Modified Eagle Medium (DMEM) (Gibco, Grand Island, NY, USA) containing 10% fetal bovine serum (Gibco). All cells were captured in the atmosphere containing 5% CO_2_ and 37 °C. The overexpression plasmids, pcDNA3.1-NR2F1-AS1 and pcDNA3.1-ZEB2 and pGPH1-sh-NR2F1-AS1 and pGPH1-si-ZEB2 were purchased from GenePharma (Suzhou, China). The mimics and inhibitors of hsa-miR-25b-3p were provided by RiboBio. Cell transfection was operated using the Lipofectamine® 3000 (Invitrogen; Thermo Fisher Scientific) according to the manufacturer's instructions.

### RNA isolation and quantitative real-time PCR (qPCR)

LncRNAs and mRNAs were extracted from the cells using Trizol reagent (Thermo Fisher Scientific). MiRNAs were extracted using an miRNA Extraction Kit purchased from Tiangen, according to the manufacturer's instructions. A PrimeScript RT Reagent kit (Takara Bio) was used to synthesize cDNA from lncRNAs and mRNA. MiRNAs were reverse transcribed using an miRNA reverse transcription kit (Takara Bio). qPCR analysis was performed in triplicate for each sample using TB Green® Fast qPCR Mix (Takara Bio Inc.), with GAPDH/U6 as the endogenous control. A two-step cycling condition was selected on Roche 480II, according to the manufacturer's instructions. Data were analyzed using the 2^-ΔΔCq^ method. The primers used are shown in Supplementary Table 1.

### Subcellular fractionation and fluorescence in situ hybridization (FISH)

Nuclear and cytoplasmic lncRNA was separated using the NE-PER™ Nuclear and Cytoplasmic Extraction Reagents (Invitrogen, Waltham, MA, USA) and quantitated using qPCR assay. For FISH assays, cells were immobilized, permeabilized, and hybridized with a 50 μL LGALS8-AS1 probe mix (RiboBio, Guangzhou, China). Cell nuclei were stained with DAPI (Sigma-Aldrich). Intracellular distribution was observed using a fluorescence microscope (Olympus IX81; Japan). The probe is shown in Supplementary Table.

### Western blotting

Total protein extracted from breast cancer cells and tissues was lysed using a lysis buffer (Beyotime, Nantong, China) with a protease inhibitor. Protein lysates were separated using 10% sodium dodecyl sulfate (SDS) gel electrophoresis and transferred to a polyvinylidene difluoride (PVDF) membrane (Millipore, USA). After blocking in 5% bovine serum albumin (BSA) for 1 h at room temperature, the membrane was incubated with primary antibodies at 4 °C overnight and then with secondary antibodies for 1 h at room temperature. The bands were imaged using an ECL assay reagent (Beyotime). The primary antibodies used were obtained from Proteintech and included anti-E-cadherin (1:500; 20874-1-AP), anti-N-cadherin (1:500; 22018-1-AP), anti-vimentin (1:1000; 10366-1-AP), anti-ZEB1 (1:1000; 21544-1-AP), and anti-ZEB2 (1:1000; 67514-1-Ig).

### Luciferase reporter assay

Sequences of the wild-type or mutant NR2F1-AS1 fragment or ZEB2 3'-UTR containing the predicted binding sites of miR-25-3p were subcloned into a psiCHECK2 dual-luciferase vector (Promega). The luciferase reporter plasmids were cotransfected into HEK293T with miR-25-3p mimics or negative control (NC) using Lipofectamine® 3000 following the manufacturer's instructions, and the transfected cells were cultured at 37 °C in a humidified incubator with 5% CO_2_ for 36 h. Luciferase signals were measured using the Dual-Glo® Luciferase Assay System (Promega) according to the manufacturer's instructions. Luciferase activity was detected using Synergy 2 Multidetector Microplate Reader (BioTek Instruments, Santa Clara, CA, USA).

### Transwell assay

An appropriate number of cells in serum-free conditioned medium were seeded into the upper chamber coated with Matrigel (BD Biosciences, San Jose, CA, USA). A complete medium was added to the lower chambers. After maintaining for 24 h at 37 °C, cells on the upper chamber were removed, and the invaded cells were treated with 4% paraformaldehyde (FD, China) and stained with crystal violet. The cells were observed and counted under a microscope.

### Wound healing assay

Cells were seeded in 6-well plates and cultured to a sub-confluent state. After starvation in serum-free DMEM (Gibco; Thermo Fisher Scientific) for 24 h, a straight wound was scratched at the bottom of the plate with a 200 μL sterile pipette tip. The cells were cultured in a serum-free medium for 24 h and observed at 0 and 24 h using an inverted light microscope. Scratch-healing (%) = (initial scratch area - final scratch area)/initial scratch area × 100.

### RNA immunoprecipitation (RIP) assay

RIP assay was performed using the Magna RIP RNA-Binding Protein Immunoprecipitation Kit, following the manufacturer's protocol (Millipore, USA). Briefly, cells were lysed in RIP Lysis buffer, the supernatant was transferred to a nuclease-free tube on ice, and the resuspended beads were added and incubated with Ago2 or IgG. The bead-bound immunoprecipitates were eluted with elution buffer, and the purified RNA fraction was analyzed using RT-qPCR.

### Statistical analysis

Data are presented as mean ± standard deviation (SD). Two groups were compared using an unpaired Student's t-test. One-way or two-way ANOVA, followed by Tukey's post-hoc test, was used to compare differences between more than two groups. The results were analyzed using GraphPad Prism 9.0. All experiments were repeated thrice. *P* < 0.05 was considered statistically significant.

## Results

### Lnc NR2F1-AS1 is upregulated and correlated with poor BC prognosis in patients

**Figure 1:**
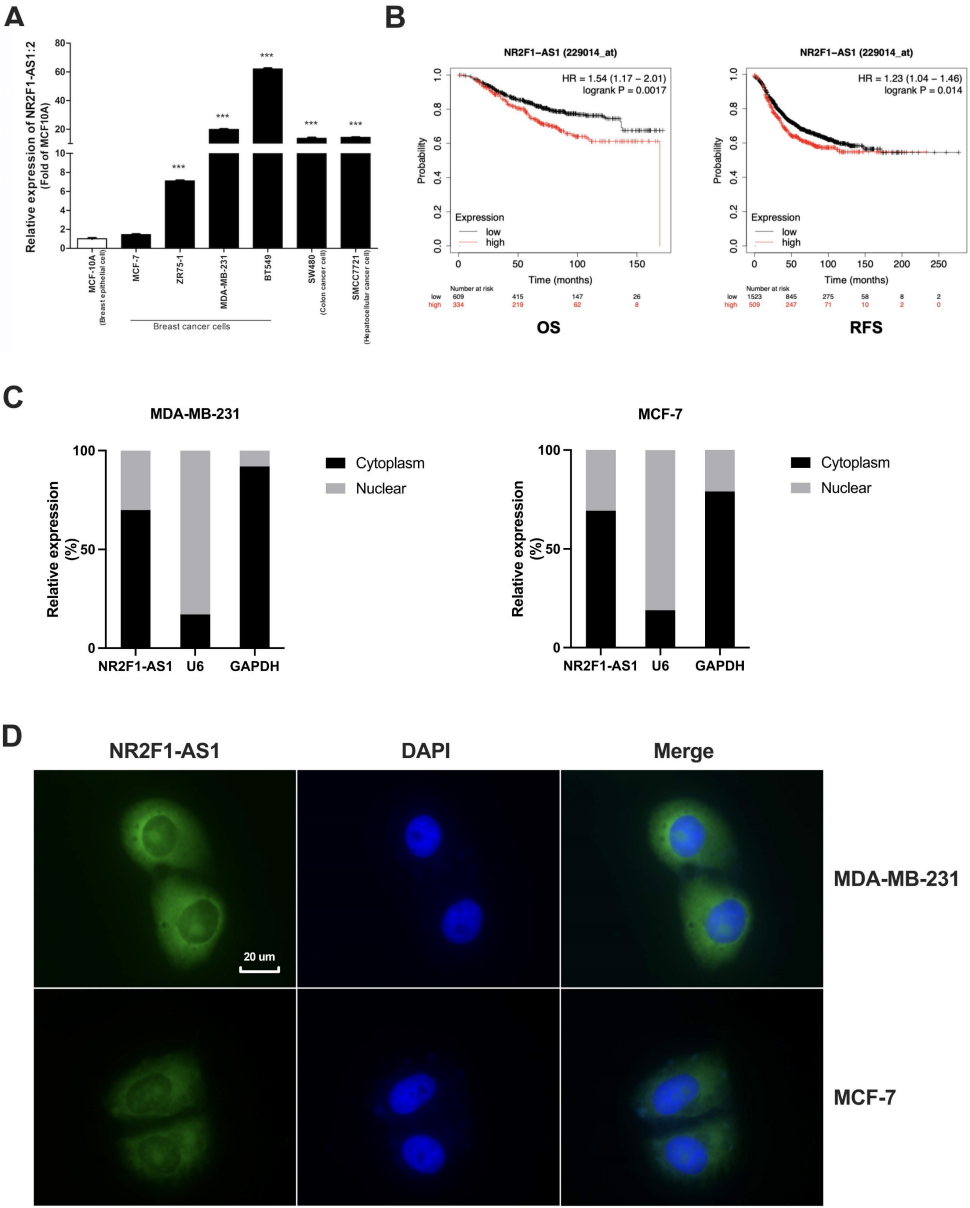
** Lnc NR2F1-AS1 upregulation is associated with poor prognosis in BC. A** RT-qPCR was used to detect lnc NR2F1-AS1 expression in normal breast cell line and BC cell line. GAPDH was the internal control (n=3 independent cultures). **B** Kaplan-Meier analysis demonstrated the relationship between lnc NR2F1-AS1 expression and overall survival (n=943) or recurrence-free survival (n=2032) of BC patients. **C** The expression level of lnc NR2F1-AS1 in the subcellular fractions of MCF7 cells and MDA-MB-231 cells was determined by qRT-PCR. GAPDH and U6 were used as cytoplasmic and nuclear markers, respectively.** D** The localization of lnc NR2F1-AS1 (Green) in MCF-7 and MDA-MB-231 cells was detected by FISH assay. Nuclei (blue) was stained by DAPI. Data are presented as the means±SEM, ***p<0.001(unpaired, two-tailed Student' s t-test).

First, we identified essential lncRNAs potentially involved in BC metastasis. Our previous lncRNA microarray (GSE201574) showed that lnc NR2F1-AS1 is upregulated in BC cells undergoing EMT. Subsequently, we validated the expression of lnc NR2F1-AS1 in normal breast cells (MCF-10A), ER positive BC cells (MCF-7, ZR-75-1), and basal-like BC cells (MDA-MB-231, BT549). The RT-qPCR results showed that lnc NR2F1-AS1 was significantly increased in BC cells, particularly in invasive BC cells (Fig. 1A). Further prognostic analysis of lnc NR2F1-AS1 suggested that high levels of lnc NR2F1-AS1 were associated with adverse overall survival (OS) and recurrence-free survival (RFS) of patients with BC, depending on Kaplan-Meier analysis (Fig. 1B). The subcellular distribution assay showed that lnc NR2F1-AS1 was mainly located in the cytoplasm (Fig. 1C), which was further validated by FISH (Fig. 1D). These findings demonstrated that lnc NR2F1-AS1 participates in BC progression.

### Inhibition of lnc NR2F1-AS1 suppressed cell migration, invasiveness, and tumor metastasis in BC

**Figure 2:**
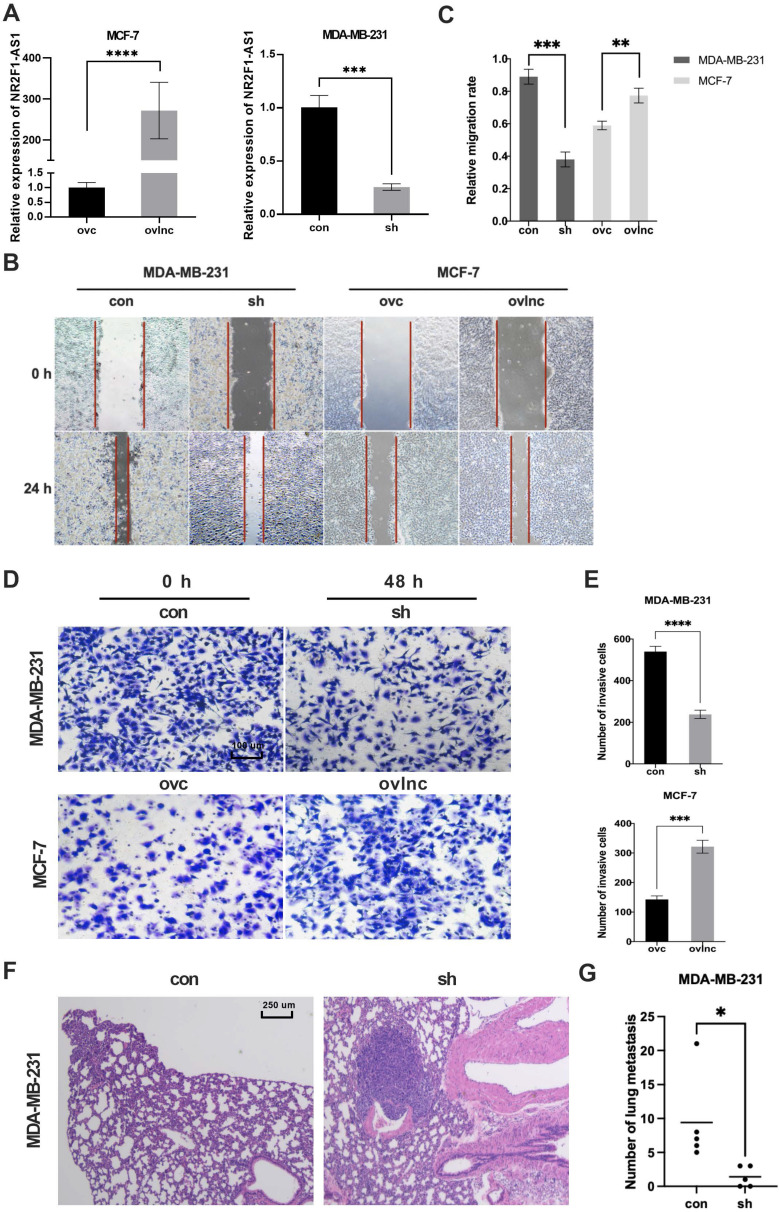
** Lnc NR2F1-AS1 promotes cell migration, invasiveness and tumor metastasis in BC. A** The expression level of lnc NR2F1-AS1 in MCF-7 and MDA-MB-231 cells after overexpression or inhibition of lnc NR2F1-AS1 were detected by RT-qPCR (n=3 independent cultures).** B, C** The effects of lnc NR2F1-AS1 overexpression or knockdown on BC cell migration were determined by wound healing assay (n=3 independent cultures). **D, E** The level of invasiveness after overexpression or inhibition of lnc NR2F1-AS1 in MCF-7 and MDA-MB-231 cells were detected by transwell assay (n=3 independent cultures).** F, G** Lung metastasis of BC in mice was measured by HE staining after lnc NR2F1-AS1 knockdown (n=5 mice per group). Data are expressed as the means±SEM, *p< 0.05, **p< 0.01, ***p< 0.001, ****p< 0.0001(unpaired, two-tailed Student's t-test for A, E, G; one-way ANOVA with Tukey' s post hoc test for C).

To investigate the functions of lnc NR2F1-AS1 in BC cells, we modulated lnc NR2F1-AS1 expression in MDA-MB-231 and MCF-7 cells using lentiviral infection. RT-qPCR showed that lnc NR2F1-AS1 expression was markedly downregulated in shlnc-infected MDA-MB-231 cells and upregulated in ovlnc-infected MCF7 cells compared to that in control (Fig. 2A). The wound healing assay revealed that lnc NR2F1-AS1 inhibition substantially reduced cell migration ability, and lnc NR2F1-AS1 overexpression significantly promoted migration potential (Fig. 2B, C). Moreover, we investigated whether lnc NR2F1-AS1 participated in cell invasion. Transwell invasion assay indicated that cell invasion was dramatically decreased in lnc NR2F1-AS1 knockdown MDA-MB-231 cells and increased in lnc NR2F1-AS1-overexpressed MCF-7 cells (Fig. 2D, E). In the animal model, the inhibition of lnc NR2F1-AS1 significantly suppressed BC lung metastasis (Fig. 2F, G). These results suggest that lnc NR2F1-AS1 suppression inhibits BC metastasis in vitro and in vivo.

### Lnc NR2F1-AS1 promotes EMT in BC cell

**Figure 3:**
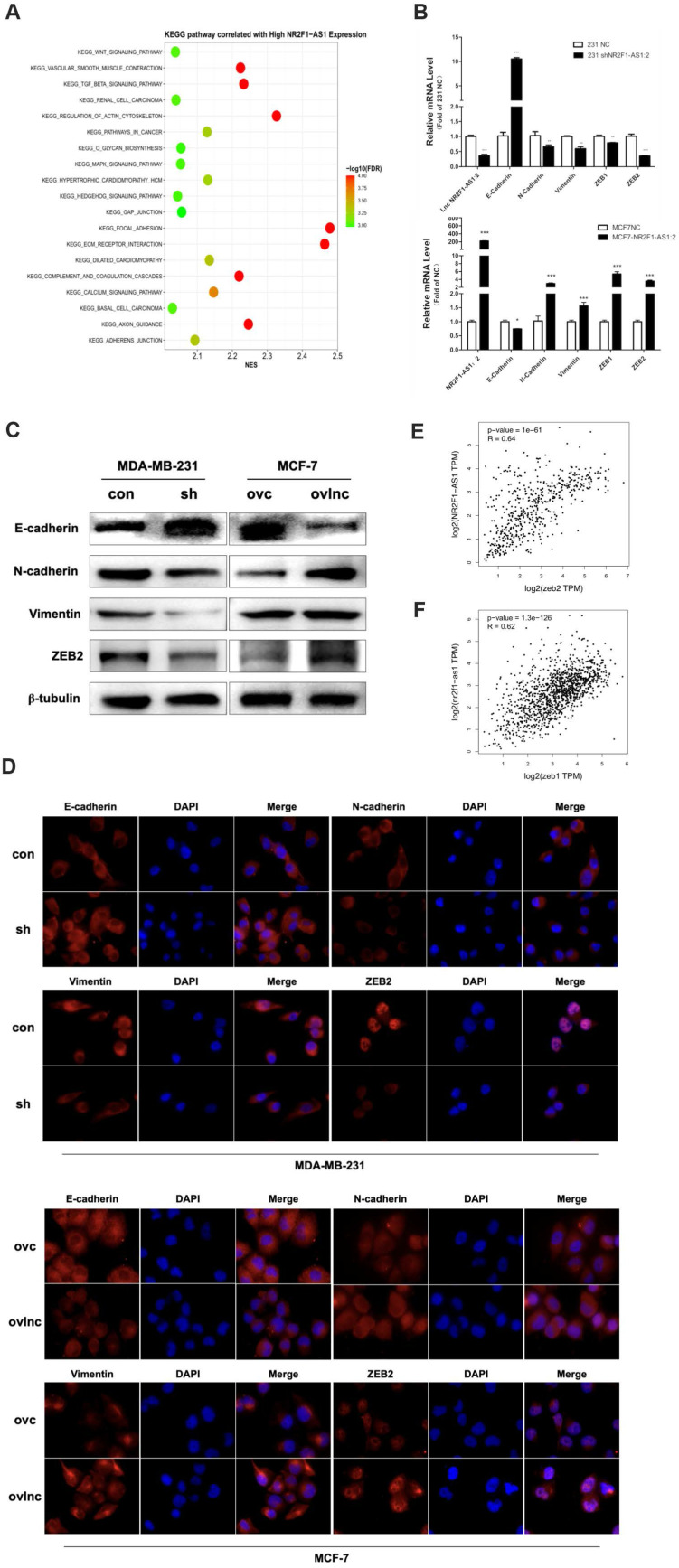
** Lnc NR2F1-AS1 promotes EMT of BC cell. A** KEGG pathway analysis of high and low lncRNA NR2F1-AS1 expression cells.** B** RT- qPCR analysis of the mRNA expression of epithelial markers (E-cadherin) and mesenchymal markers (N-cadherin, Vimentin, ZEB2) upon LncRNA NR2F1- AS1 overexpression or knockdown (n=3 independent experiments). **C, D** Protein level of epithelial markers and mesenchymal markers in MCF-7 and MDA-MB-231 cells after overexpression or inhibition of lnc NR2F1-AS1 were detected by Western blooting and immunofluorescence.** E, F** Pearson's correlation analysis was used to explore the association between expression of lnc NR2F1-AS1 and ZEB1 or ZEB2. Data are presented as the means±SEM, *p< 0.05, **p< 0.01, ***p< 0.001(one-way ANOVA with Tukey' s post hoc test).

To further investigate how lnc NR2F1-AS1 regulates BC metastasis, we performed a KEGG analysis. Lnc NR2F1-AS1 was closely related to focal adhesion and the transforming growth factor beta (TGF-β) pathway (Fig. 3A). Because EMT is an essential downstream of the TGF-β pathway 14, 15, we investigated whether lnc NR2F1-AS1 modulation affected EMT in BC cells. The knockdown of lnc NR2F1-AS1 upregulated the expression of epithelial markers (E-cadherin) and downregulated the expression of mesenchymal markers (N-cadherin, vimentin, and ZEB2). In contrast, lnc NR2F1-AS1 overexpression decreased epithelial marker expression and increased mesenchymal marker expression (Fig. 3B-D). Moreover, lnc NR2F1-AS1 expression was positively associated with ZEB1 and ZEB2 expression in TCGA database analysis (Fig. 3E, F). These findings suggest that lnc NR2F1-AS1 promotes BC metastasis by inducing EMT.

### Lnc NR2F1-AS1 acts as a molecular sponge for miR-25-3p in BC cells

**Figure 4:**
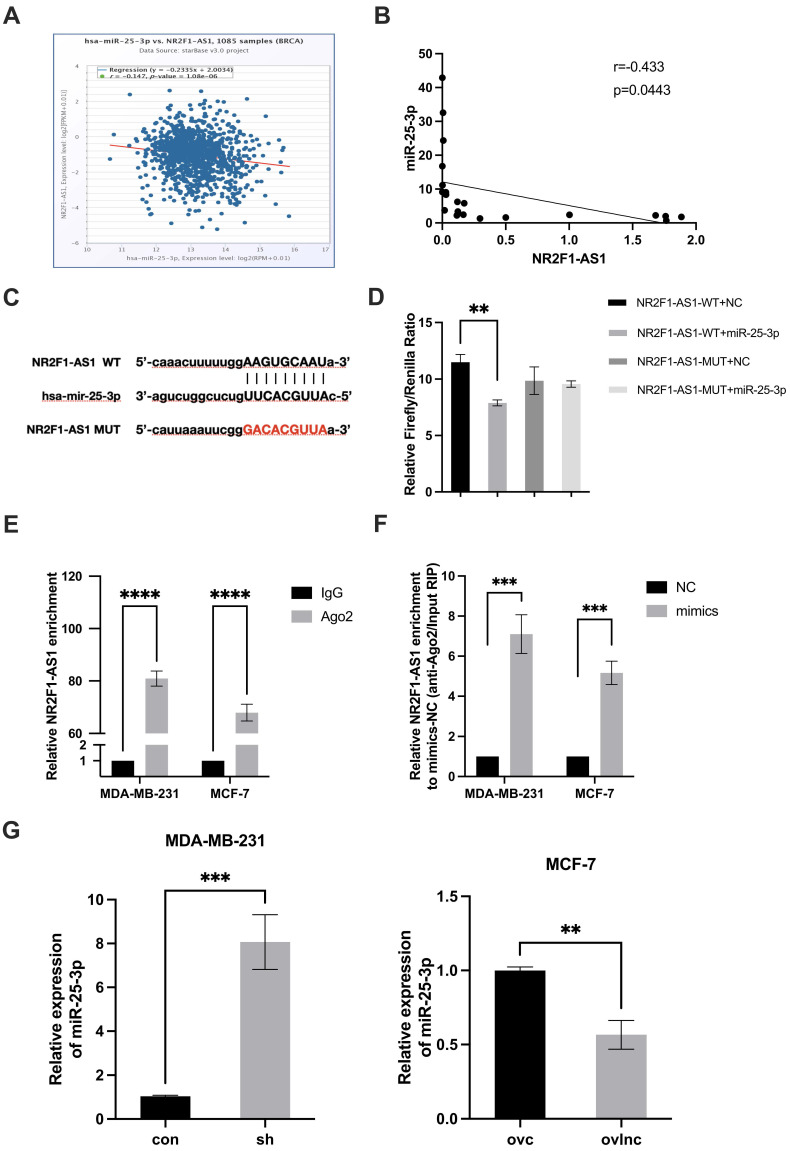
** lnc NR2F1-AS1 functions as a miR-25-3p sponge in BC cells. A, B** Correlation analysis was applied to evaluate the relationship between expression of lnc NR2F1-AS1 and miR-25-3p in BC tissues from TCGA database (n=1085) and our cohort (n=22). **C** The putative binding site for miR-25-3p in lnc NR2F1-AS1 and mutant sequences of the binding sites were shown. **D** Luciferase assays in HEK293T cells cotransfected with WT or mutant lnc NR2F1-AS1 and miR-25-3p NC or miR-25-3p mimic (n=3 independent experiments). **E** Anti-AGO2 RIP was performed in HEK293T cells, followed by RT-qPCR to examine the expression of lnc NR2F1-AS1 associated with AGO2 (n=3 independent experiments). **F** RT-qPCR was used to determine the effect of miR-25-3p NC or miR-25-3p mimic on the expression of lnc NR2F1-AS1 associated with AGO2. (n=3 independent experiments). **G** RT-qPCR analysis of the mRNA expression of miR-25-3p in lnc NR2F1-AS1 overexpressed MCF-7 cells and lnc NR2F1-AS1 inhibited MDA-MB-231 cells (n=3 independent experiments). Data are expressed as the means±SEM, **p< 0.01, ***p< 0.001, ****p< 0.0001(unpaired, two-tailed Student' s t-test for G; one-way ANOVA with Tukey' s post hoc test for D, E, F).

Cytoplasmic lncRNAs can act as ceRNAs to modulate RNA expression and activity 16, 17. According to our data, lnc NR2F1-AS1 was mainly distributed in BC cell cytoplasm (Fig. 1C, D). Thus, we presumed that lnc NR2F1-AS1 acts as a ceRNA to prevent miRNAs from degrading their target mRNAs. Using bioinformatics software, we identified miR-25-3p as a potential target of lnc NR2F1-AS1. Additionally, lnc NR2F1-AS1 was inversely correlated with miR-25-3p in TCGA database and our cohort (Fig. 4A, B). A dual luciferase reporter assay was performed to confirm binding potential. Overexpression of miR-25-3p decreased the luciferase activity of the pmirGLO-NR2F1-AS1-WT vector but not of the pmirGLO-NR2F1-AS1 mutant vector (Fig. 4C, D). Furthermore, endogenous lnc NR2F1-AS1 was pulled down by the AGO2 antibody, and overexpression of miR-25-3p mimics significantly upregulated lnc NR2F1-AS1 binding to the AGO2 antibody in the RNA immunoprecipitation assay (Fig. 4E, F). Furthermore, lnc NR2F1-AS1 inhibition promoted miR-25-3p expression, whereas lnc NR2F1-AS1 overexpression suppressed miR-25-3p expression (Fig. 4G). These data demonstrated that miR-25-3p is an inhibitory target of lnc NR2F1-AS1 in BC.

### Lnc NR2F1-AS1 promotes ZEB2 expression via miR-25-3p suppression

**Figure 5:**
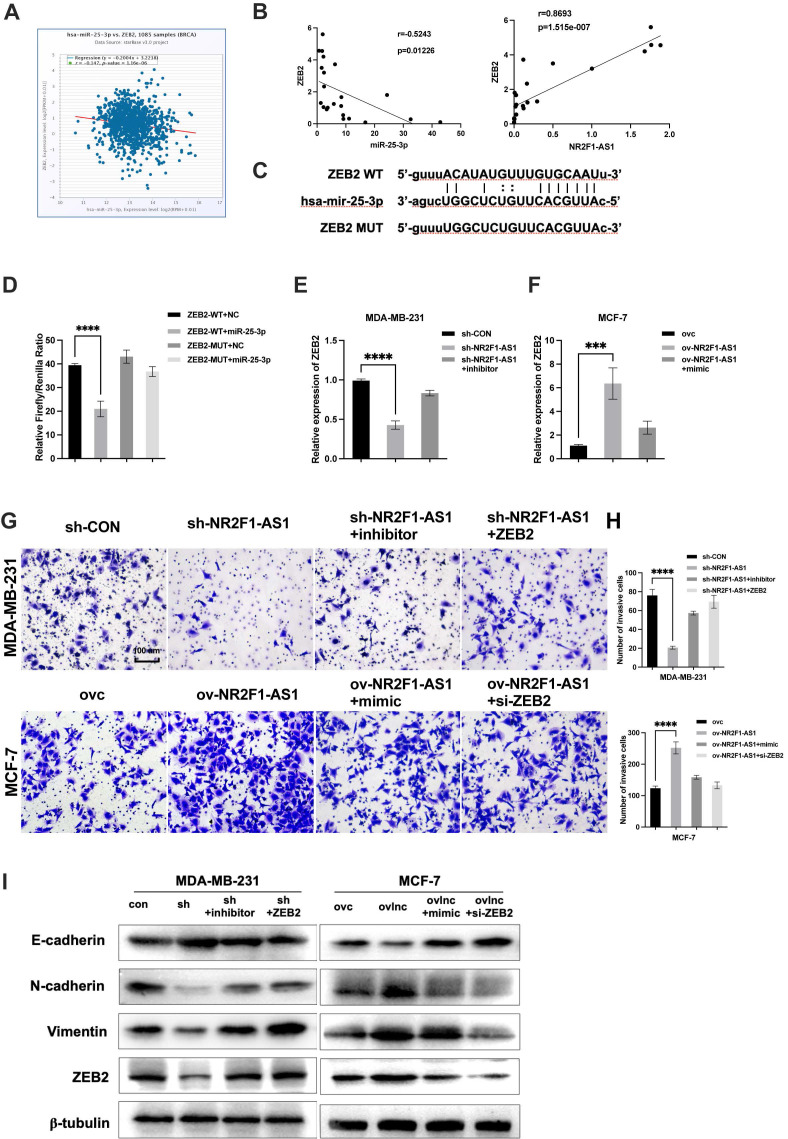
** Lnc NR2F1-AS1 upregulates ZEB2 expression via inhibition of miR-25-3p. A** Correlation analysis was applied to assess relationship between ZEB2 and miR-25-3p in BC tissues from TCGA database. **B** The association between ZEB2 and lnc NR2F1-AS1 or miR-25-3p were determined by Pearson's correlation analysis.** C** The putative binding site for miR-25-3p in ZEB2 and mutant sequences of the binding sites.** D** Luciferase reporter assays in HEK293T cells cotransfected with WT or mutant ZEB2 and miR-25-3p NC or miR-25-3p mimic (n=3 independent experiments). **E, F** The effects of ov lnc NR2F1-AS1 and miR-25-3p mimic cotransfection or sh lnc NR2F1-AS1 and miR-25-3p inhibitor cotransfection on ZEB2 expression were detected by qPCR (n=3 independent experiments). **G, H** The effects of ov lnc NR2F1-AS1 and miR-25-3p mimic cotransfection or sh lnc NR2F1-AS1 and miR-25-3p inhibitor cotransfection on cell invasiveness were measured by transwell assay (n=3 independent experiments). **I** Western blotting was used to detect protein level of epithelial markers and mesenchymal markers in MCF7 cells cotransfected with miR-25-3p mimic or siZEB2 and ov lnc NR2F1-AS1 and MDA-MB-231 cotransfected with miR-25-3p inhibitor or ZEB2 and shlnc NR2F1-AS1. Data are presented as the means±SEM, ***p< 0.001, ****p< 0.0001(one-way ANOVA with Tukey' s post hoc test).

Using the StarBase database, we identified ZEB2 as a potential target of miR-25-3p. ZEB2 is a key transcription factor regulating EMT in BC 18, 19. However, the relationship between lnc NR2F1-AS1/miR-25-3p and ZEB2 in BC remains poorly understood. ZEB2 expression was negatively associated with miR-25-3p expression in TCGA database (Fig. 5A). Additionally, ZEB2 was inversely correlated with miR-25-3p or lnc NR2F1-AS1 in our cohort (Fig. 5B). Thus, ZEB2 was selected as a miR-25-3p target for further investigation. The predicted binding site of miR-25-3p on the ZEB2 mRNA is shown in Figure 5C. Our luciferase assay indicated that miR-25-3p overexpression reduced the luciferase activity of the WT ZEB2 vector but not of the mutant ZEB2 vector (Fig. 5D). Moreover, the mRNA level of ZEB2 was downregulated by lnc NR2F1-AS1 inhibition and reversed by the miR-25-3p inhibitor. Furthermore, ZEB2 mRNA expression was increased by lnc NR2F1-AS1 overexpression and decreased by the miR-25-3p mimic (Fig. 5E). Finally, transwell invasion and WB assays showed that miR-25-3p inhibition or ZEB2 overexpression significantly promoted BC invasion, and EMT capacities were suppressed by lnc NR2F1-AS1 knockdown. MiR-25-3p overexpression or ZEB2 inhibition dramatically decreased lnc NR2F1-AS1 overexpression-induced BC invasion and EMT (Fig. 5F-H). These results suggest that lnc NR2F1-AS1 functions as a ceRNA to increase ZEB2 levels by competitively binding to miR-25-3p and ultimately enhance BC cell EMT, migration, and invasion.

## Discussion

Therapeutic methods effective in patients with metastatic BC are limited. Thus, there is a clinical need for comprehensively understanding the specific mechanisms behind BC metastasis 20, 21. LncRNAs have been identified as essential regulators of cancer progression. The functions and modulation of lncRNAs in the identification of potential targets for BC treatment have been revealed 22-24. Lnc NR2F1-AS1, an antisense lncRNA of NR2F1, is also known as COUP Transcription Factor I. High levels of lnc NR2F1-AS1 promote gastric cancer and hepatocellular carcinoma progression 25, 26. Lnc NR2F1-AS1 was up-regulated in the dormant mesenchymal-like breast cancer stem-like cells, and functionally promotes tumor dissemination but reduces proliferation in lungs 27. In the current study, lnc NR2F1-AS1 was upregulated in BC tissues and cells, and high lnc NR2F1-AS1 expression was closely related to poor prognosis in patients with BC. Functional studies revealed that the inhibition of lnc NR2F1-AS1 suppressed migration, invasion, and EMT of BC cells in vitro and lung metastasis in vivo, suggesting a carcinogenic role of lnc NR2F1-AS1 in BC.

The subcellular distribution is crucial for understanding the biological functions of lncRNAs. Cytoplasmic lncRNAs are involved in post-transcriptional gene regulation, serving as ceRNAs and preventing downstream target mRNA from degradation 28-30. Using cell cytoplasmic/nuclear fractionation and FISH assays, we found that lnc NR2F1-AS1 was mainly located in the cytoplasm of BC cells. Therefore, we presumed that lnc NR2F1-AS1 might act as a miRNA sponge in BC. MiR-25-3p was previously considered a tumor suppressor in colorectal and cervical cancers 31, 32. MiR-25-3p expression was negatively associated with lnc NR2F1-AS1 in BC tissues from TCGA database and our cohort. Next, we identified the specific binding sites between miR-25-3p and lnc NR2F1-AS1 using three online bioinformatics tools (RNAhybrid, StarBase v2.0, and TargetScan), which were further validated by RIP and dual luciferase reporter assays. Moreover, miR-25-3p significantly reversed the modulatory effect of BC invasion and EMT induced by lnc NR2F1-AS1 inhibition or overexpression. Our results confirmed the antitumor effect of miR-25-3p in cancer and investigated its functions in BC EMT.

The transcription factors ZEB1 and ZEB2, key regulators of cell invasion and EMT, have been investigated in several solid cancers, including BC. ZEB1 and ZEB2 exert their EMT-inducing functions through transcriptional repression of the E-cadherin gene 33-35. Our data showed that lnc NR2F1-AS1 upregulated ZEB1 and ZEB2 expression in BC. Furthermore, we confirmed that ZEB2 is a direct target of miR-25-3p. Lnc NR2F1-AS1 positively modulated ZEB2 expression by sponging miR-25-3p. The biological effects induced by lnc NR2F1-AS1 inhibition were reversed by ZEB2 overexpression in BC cells, highlighting the lnc NR2F1-AS1/miR-25-3p/ZEB2 axis in promoting BC cell migration, invasion, and EMT. In addition, we investigated the association between ZEB1 and miR-25-3p and obtained negative results (data not shown). Thus, lnc NR2F1-AS1 may upregulate ZEB1 expression through other mechanisms, which requires further investigation.

## Conclusions

In summary, we identified a novel lnc NR2F1-AS1, which was upregulated in BC and correlated with adverse prognosis in patients with BC. Lnc NR2F1-AS1 functioned as a sponge for miR-25-3p and contributed to BC EMT and metastasis by activating ZEB2 expression. Our findings demonstrate the lnc NR2F1-AS1/miR-25-3p/ZEB2 axis as a potential therapeutic target for BC metastasis.

## Supplementary Material

Supplementary figures and tables.

## Figures and Tables

**Figure 6:**
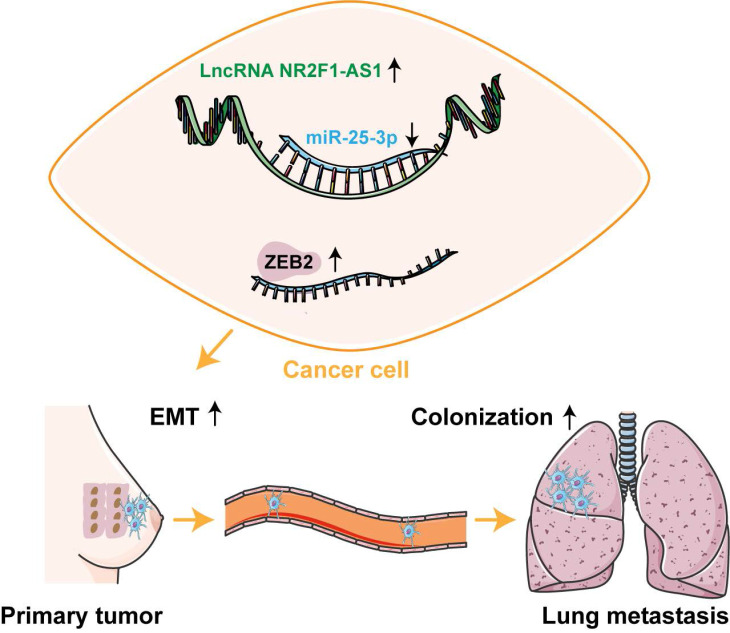
Schematic diagram of lnc NR2F1-AS1 function in BC.

## Abbreviations

BC: breast cancer
EMT: epithelial-to-mesenchymal transition
MET: mesenchymal-to-epithelial transition
lncRNA: long non-coding RNA
ceRNA: competing endogenous RNA
TCGA: The Cancer Genome Atlas
miRNA: microRNA
KEGG: Kyoto Encyclopedia of Genes and Genomes
ATCC: American Type Culture Collection
DMEM: Dulbecco's Modified Eagle Medium
qPCR: quantitative real-time polymerase chain reaction
FISH: fluorescence in situ hybridization
SDS: sodium dodecyl sulfate
PVDF: polyvinylidene difluoride
BSA: bovine serum albumin
NC: negative control
RIP: RNA immunoprecipitation
SD: standard deviation
OS: overall survival
RFS: recurrence-free survival
TGF-β: transforming growth factor beta
