# Supplementary material for: Lnc NR2F1-AS1 Promotes Breast Cancer Metastasis by Targeting the MiR-25-3p/ZEB2 Axis
Source: Int J Med Sci. 2023 Jul 24;20(9):1152–62. doi: 10.7150/ijms.86969 (PMC10416723; doi:10.7150/ijms.86969)
Supplement: Supplementary file 1 — Supplementary figures and tables. [file ijmsv20p1152s1.pdf]

Supplementary table 1 Primers for RT-qPCR

Genes	Primers
ZEB2	5′- CACACACATACACAGAAAGGA-3′ (forward)<br>5′- ATAACAGGAGGCATAGCATT-3′ (reverse)
GADPH	5′- GTCTCCTCTGACTTCAACAGCG-3′ (forward)<br>5′- ACCACCCTGTTGCTGTAGCCAA-3′ (reverse)
U6	5′- CTCGCTTCGGCAGCACATATACT-3′ (forward)<br>5′-ACGCTTCACGAATTTGCGTGTC-3′ (reverse)
miR-25-3p	5′- CATTGCACTTGTCTCGGTCTGA-3′ (forward)<br>5′- GCTGTCAACGATACGCTACGTAACG-3′ (reverse)
NR2F1-AS1	5′- TTGAGGCACTTCTTGAGG-3′ (forward)<br>5′- CCGCAGGAACTTAACTTAC-3′ (reverse)

Probe sequence for NR2F1-AS1

CAUCAAUAGAGAUGAGCUGCAAGUUGUUGUUCCAUAGUUGACAUAGGC
UUCCUGAUCUAGCCAAUUCUAUUGCCAAAGCUCCCCAGUGAAUGGCAGC
UUACAUCACGGCAUGGUAGCUACCAUAAGGAUGCAGCUCUGGGGCUCC
UCUUGCAUAUGGAAAUAAGGAAAUUUUAAUAGAAAUAUUGAGAACAUC
UGCUGCAACCUGUGAUGCAGGCCCACUGCCACCGCCAUUCAUCCUGGUU
AAUAUUGUGGUCACGGAGAAAACAGGUUCAUCAUGGGCUAGUUUCAUU
UAUCCACACCUUUUCUCUUGAGUUUCAUCUACCUGCUGC

Sequences of RNAi

Construct	Sequence

shNR2F1-AS1	5′-ccggAAGGTGTGGATAAATGAAACTctcgagAGTTTCATTTATCCACACCTTtttttg-3′
siZEB2	5’- GGACACAGGUUCUGAAACA-3’

Figure S1

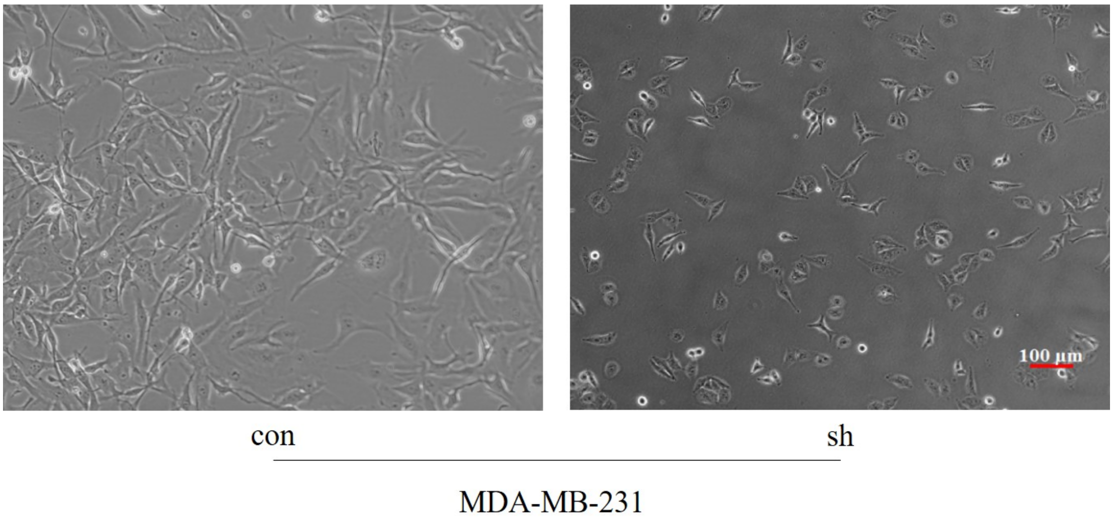

Figure S1 The morphological change of MDA-MB-231 cells.

Figure S2

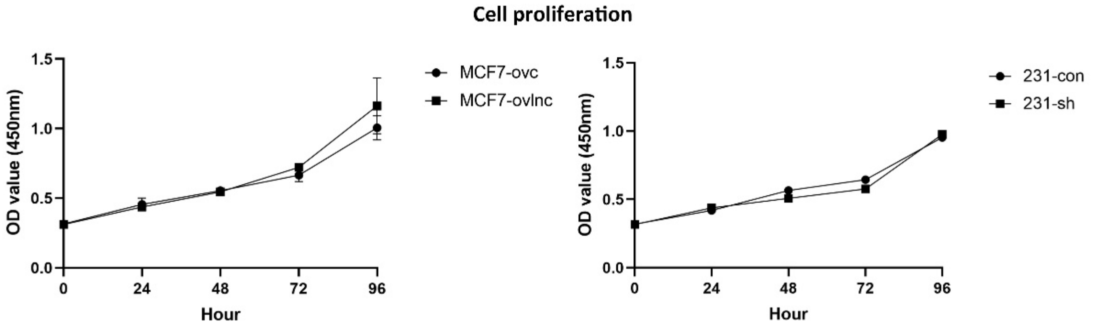

Figure S2 The growth curve of CCK-8 assay showed that overexpression of lnc NR2F1-AS1 or knockdown of lnc NR2F1-AS1 didn’t influence the proliferation of MCF-7 ($p = 0.165$) and MDA-MB-231 ($p = 0.487$) cells, respectively.
